# Data Augmentation in Classification and Segmentation: A Survey and New Strategies

**DOI:** 10.3390/jimaging9020046

**Published:** 2023-02-17

**Authors:** Khaled Alomar, Halil Ibrahim Aysel, Xiaohao Cai

**Affiliations:** School of Electronics and Computer Science, University of Southampton, Southampton SO17 1BJ, UK

**Keywords:** data augmentation, deep learning, convolutional neural networks, image processing, segmentation, classification

## Abstract

In the past decade, deep neural networks, particularly convolutional neural networks, have revolutionised computer vision. However, all deep learning models may require a large amount of data so as to achieve satisfying results. Unfortunately, the availability of sufficient amounts of data for real-world problems is not always possible, and it is well recognised that a paucity of data easily results in overfitting. This issue may be addressed through several approaches, one of which is data augmentation. In this paper, we survey the existing data augmentation techniques in computer vision tasks, including segmentation and classification, and suggest new strategies. In particular, we introduce a way of implementing data augmentation by using local information in images. We propose a parameter-free and easy to implement strategy, the random local rotation strategy, which involves randomly selecting the location and size of circular regions in the image and rotating them with random angles. It can be used as an alternative to the traditional rotation strategy, which generally suffers from irregular image boundaries. It can also complement other techniques in data augmentation. Extensive experimental results and comparisons demonstrated that the new strategy consistently outperformed its traditional counterparts in, for example, image classification.

## 1. Introduction

Deep neural networks, like convolutional neural networks (CNNs), have been used in computer vision with numerous research applications, such as action recognition [1,2], object detection and localisation [3,4], face recognition [5], and image characterisation [6]. They have achieved superior performance against conventional approaches in many challenging computer vision tasks [7]. Nevertheless, their shortcomings, such as large-scale data requirements, long training time, overfitting, and performance slumps upon data scarcity, may hinder their generalisation and effectiveness [8,9].

**Figure 1:**
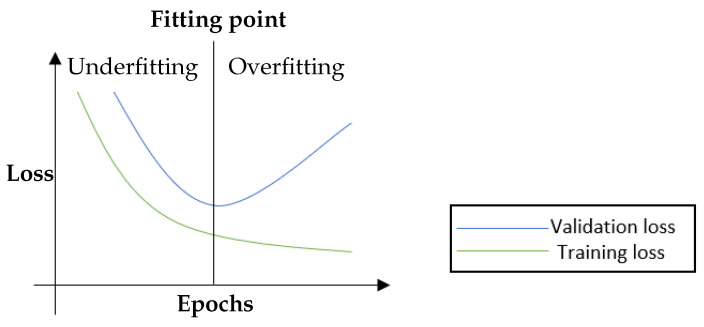
An illustration of the training and validation loss curves. Training and validation losses decrease simultaneously until the fitting point. After that, the validation loss begins to rise while the training loss is still decreasing, i.e., the so-called overfitting. Overfitting is associated with good performance on the training data but poor generalisation to the validation/test data (cf. underfitting is associated with poor performance on the training data and poor generalisation to the validation data) [12].

**Figure 2:**
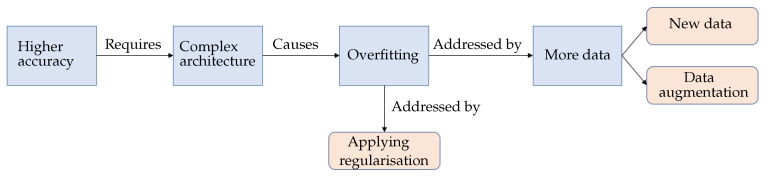
Diagram illustrating the overfitting problem and its well-known solutions.

The fruitful results presented by the CNN models encourage researchers to pursue higher accuracy models. These results are generally achieved by building more complex architectures [10]. Note that model complexity is often described by the number of trainable parameters. The more trainable parameters a model has, the more complex it is. More specifically, model complexity may also be defined in terms of the number of layers (i.e., non-linearity) and the number of neurons (e.g., filters) in individual layers. On the other hand, in supervised learning, data complexity can be determined according to the inter-class multiplicity (i.e., different classes) in addition to the intra-class differences. In general, the complex of the data and the model needed is proportional. If the training data is insufficient, complex models may be susceptible to the issue of memorising the training data. It is also well known that deep neural networks prevail partly because of the availability of high volume data. The networks can easily memorise data points due to their complex structure. However, the increasing complexity of the model architectures with insufficient data could exacerbate the shortcomings of CNN models [11]. One of the most apparent issues when adopting complex CNN models is the overfitting problem [12], which can be described as the performance difference between the training and validation/test stages, where the model loses its ability to generalise. Overfitting generally occurs when a model is either too complex for the data or the data itself is insufficient [13]. Figure 1 shows an example of the loss curve of an overfit model. Although the training accuracy and validation accuracy improved concurrently during the early stages of training, they diverged after a certain point, where the model started losing its generalisation ability [12]. Strategies like reducing the model complexity, applying regularisation, and/or acquiring more extensive data volumes have been considered to mitigate the overfitting issue in deep learning models, see Figure 2.

Regularisation techniques are implemented at the model architectural level [14,15], such as dropout [16], ridge regression ($\ell_{2}$ regularisation) [17], and Lasso regression ($\ell_{1}$ regularisation) [18]. The main objective of these techniques is to reduce the complexity of a neural network model during training, which is considered the main reason behind overfitting, especially when the model is trained on small datasets. Other techniques, like batch normalisation and transfer learning, may speed up the training process and also have an impact on preventing overfitting [19,20]. These techniques could be regarded as byproducts of the constant competition in the pursuit of higher performance by innovating new complex deep neural architectures, such as VGG-16 [21], ResNet [22], Inception-V3 [23] and DenseNet [24]. These models, in fact, aim to achieve higher accuracy on large datasets like Imagenet [25], which has over 14 million images [25]. However, when applying these models to small-scale applications with small datasets, they usually suffer from poor generalisation and overfitting, indicating the necessity of developing methods to reduce their complexity.

Data augmentation methodology encompasses a broader range of techniques that function at the data level, rather than at the model architectural level. It can help deep learning models perform better by artificially creating different and diverse samples with balanced classes for the training dataset. When the dataset is sufficient in terms of quantity and quality, a deep learning model performs better and more accurately. In other words, the training data must fulfil two requirements, i.e., adequate diversity and size, both of which can be achieved by data augmentation [26].

**Figure 3:**
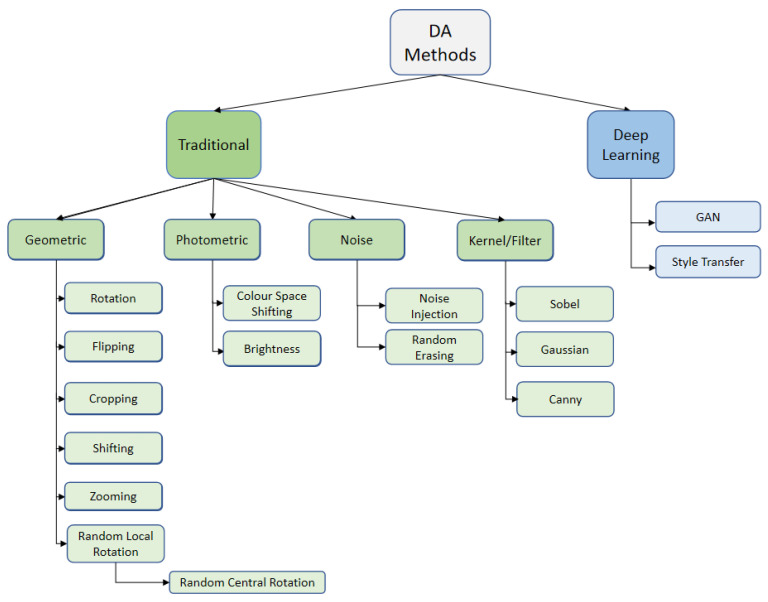
Data augmentation (DA) taxonomy.

Data augmentation can be categorised based on the intended purpose of applying it (i.e., increasing training dataset size and/or diversity), or it can be categorised based on the problems. The following are examples of the latter: the random erasing technique was proposed to address the occlusion problem [8]; rotation and flipping were supposed to partially resolve the viewpoint problem [27,28,29]; brightness was used to address the change in lighting [30]; and cropping and zooming were used to address the scaling and background issues. In particular, the most popular categorisation of data augmentation divides it into deep learning-based data augmentation and traditional data augmentation [10], which is further divided into geometric, photometric, and noise data augmentation, see Figure 3. For reviews on the deep learning approaches for data augmentation, see e.g., [31,32].

Several studies evaluating the efficacy of data augmentation have utilised standard academic image datasets to assess results. For example, MNIST, CIFAR-10, CIFAR-100 and ImageNet are four commonly used datasets [10,33,34,35]. Note that some of these datasets, especially ImageNet, are considered “big data” [36] and may not require data augmentation techniques to further increase their size. To simulate data scarcity challenges, many experiments testing data augmentation techniques limit themselves to small subsets of the original large datasets [34]. It is worth emphasising that data augmentation techniques may also be used to improve the data diversity, except for the data quantity.

**Figure 4:**
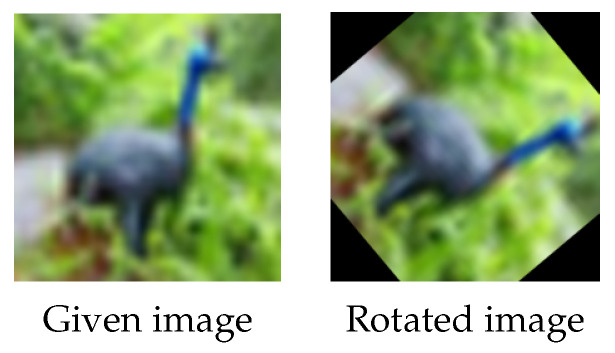
Traditional rotation. Left and right: the given image (from the CIFAR-10 dataset) and the rotated image with a randomly rotated angle. Black areas appear in the corners of the rotated image and the corners in the given image are cut off in the rotated image.

**Figure 5:**
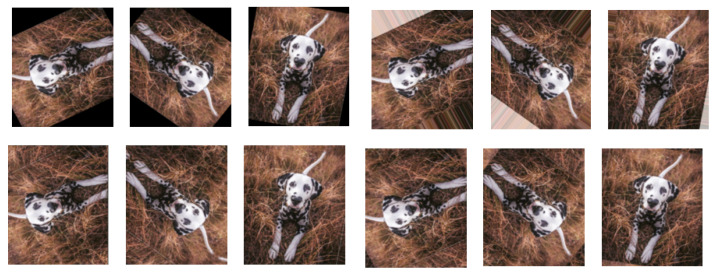
Data augmentation by different types of rotation techniques. The left three figures in the first row show the “constant” technique, i.e., the traditional rotation (TR), resulting in black areas around the boundary. The RNR technique is used in the right three figures of the first row. The first three figures of the second row give the results of filling up the black areas by using RRR, and the right three figures use RWR. For each technique, three random angles were selected for rotation.

This survey mainly focused on recent articles that used data augmentation techniques in image classification and segmentation, regardless of the data augmentation category, models, or datasets used in the studies. To the best of our knowledge, there are few surveys in the fields of data augmentation in image classification and segmentation. Another main contribution of this article is that we propose a new geometric data augmentation technique, which can complement the current data augmentation strategies. It is well known that traditional rotation is one of the most commonly used geometric data augmentation techniques, see Figure 4. It, however, has drawbacks; for example, the loss of a significant amount of pixel information when rotating. It is noticeable that rotating a square-shaped image in a circular trajectory produces black patches at the boundaries, which do not accurately reflect the original data and may affect the final augmentation performance. Filling these black patches with modified pixel values via the wrap, constant, reflection, and/or nearest rotation techniques was a common solution to this issue (see Figure 5). In this study, we suggest exploiting local information in images and propose conducting rotation randomly and locally to address the limitations of the traditional rotation. We named our method “*random local rotation*” (RLR). RLR rotates an image’s internal circular region by selecting random location, area and angle, which is easy to implement. Rotation performed in a local manner avoids forming black regions near image boundaries. Moreover, this method could also improve the data diversity. Extensive experiments demonstrated its superior performance compared to its counterpart, i.e., the traditional rotation technique.

The remainder of this article is organised as follows. Section 2 and Section 3 recall the most common traditional data augmentation methods and the most common deep learning-based data augmentation methods, respectively. Section 4 reviews some recent research in image classification and segmentation utilising data augmentation for performance enhancement. Section 5 and Section 6 present our proposed data augmentation method and the experimental results validating its promising performance. We conclude our study in Section 7.

## 2. Traditional Data Augmentation Techniques

This section briefly recalls the most commonly used traditional data augmentation approaches.

### 2.1. Geometric Transformations

Basic geometric operations. like flipping, cropping and random rotation, are still sought-after techniques to augment data. They generally increase the data size to improve data diversity, and are fairly easy to apply, see below for more detailed description.

**Figure 6:**
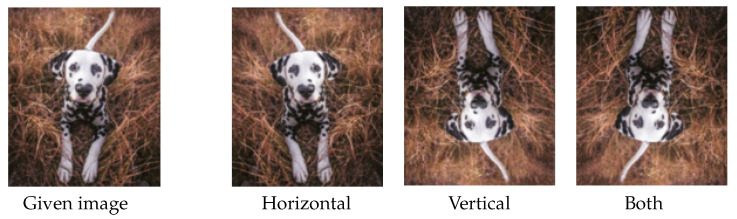
Data augmentation by flipping. Images from left to right represent the given image, horizontally flipped image, vertically flipped image and the image flipped horizontally and vertically, respectively.

**Flipping.** The term flipping refers to the process of flipping images horizontally or vertically or both, see Figure 6. The most commonly used flipping is horizonal flipping, since it is more realistic. For example, a cat versus dog dataset may include all the dog images heading to the left from the spectator view. Not surprisingly, the trained model may suffer from misclassifying dogs heading to the right. The best way to alleviate this problem is to collect more training images that include as many different views as possible. When collecting more images is difficult, flipping may directly solve this type of problem.

Flipping is one of the most intuitive strategies to increase data size or diversity. However, it may be inappropriate when the data has unique properties. For example, considering the concept of label safety, discussed in [10], asymmetric or direction sensitive data, such as letters or digit numbers, cannot use the flipping strategy since it results in inaccurate labels, or even opposite labels.

**Cropping.** Cropping is a basic augmentation technique that randomly crops a part of the given image and then resizes the cropped part back to a certain size. As training data may include samples of different sizes, cropping images to a certain size is a widely used step before training [10,37].

**Figure 7:**
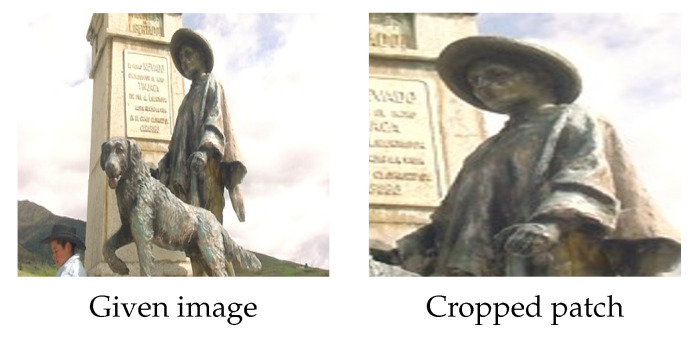
Data augmentation by cropping. Left and right: the given image (from ImageNet) labelled as “Dog” and the cropped patch. It is clear that the “Dog” is no longer visible in the cropped patch.

It is worth mentioning that cropping may generate samples with incorrect labels. For example, images containing more than one object, which are labelled according to the object with dominant size, may experience a problem when using the cropping technique. In such a case, it is possible to crop an area of the given image that has more details of the accompanying object, rather than the dominant object, see Figure 7. The conventional strategy for training modern state-of-the-art architectures is to crop patches as small as 8% of the given image and label them the same as the given image [38]. This frequently results in incorrect labelling in the augmented data, as in the example shown in Figure 7.

**Rotation.** Rotation is a simple geometric data augmentation technique. The images are rotated by a specified angle, and the newly created images are used alongside the originals as training samples. The disadvantage of rotation is that it may result in information loss at the image boundary, see Figure 4 and the first row in Figure 5. There are several possible solutions, e.g., random nearest neighbor rotation (RNR), random reflect rotation (RRR) and random wrap rotation (RWR), to fix the boundary problem of the rotated images. In particular, the RNR technique repeats the nearest pixel values to fill in the black areas, while the RRR technique employs a mirror-based approach and the RWR technique uses the periodic boundary strategy to fill in the gaps; see Figure 5 for an example.

These geometric data augmentation techniques have been shown to be highly effective in improving diversity and increasing data quantity. For example, Mash et al. [39] used a fine-grained dataset of ten classes to test a variety of geometric augmentation methods for the task of aircraft classification. Cropping, rotating, rescaling, polygon occlusion, and a combination of these techniques were all tested. The cropping technique combined with occlusion achieved the highest improvement, i.e., increasing the task performance by 9% against the benchmark result. Their study, however, did not examine photometric data augmentation strategies (see below).

### 2.2. Photometric Transformations

A different type of traditional transformation is to change pixels’ values rather than their positions. This approach includes different techniques, such as changing brightness, contrast, and/or colours.

Typically, a digital image is encoded as a tensor of three dimensions, i.e., height × width × colour channels. The difference between different colour representation schemes lies in the channel part of the tensor. For example, the RGB colour representation scheme uses a combination of three colour channels (i.e., red, green and blue) to represent individual pixels. Manipulating these individual colour channels is a very basic technique in colour augmentation [10]. For example, an image can be swiftly transformed into its representation in one colour channel if the others are set to black.

In addition to the RGB colour space, there are many other colour spaces. For example, the HSL colour representation scheme combines hue, saturation and lightness to represent individual pixels [40]. A hue is a single pigment that has no tint or shade. Saturation refers to colour intensity and lightness refers to how light a colour is. HSL is user-friendly since it is convenient to see how a particular colour appears using different values for these three attributes. Please refer to e.g., [40,41] for different colour spaces. Transferring from one colour space to another can be a useful technique for data augmentation.

**Figure 8:**
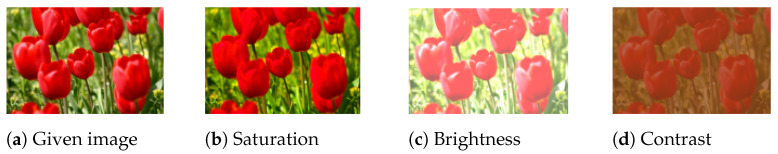
Data augmentation by colour jittering. (**a**–**d**) represent the given image, and the augmented images by manipulating the colour saturation, brightness and contrast, respectively.

Colour jittering is a photometric data augmentation technique that employs either random colour manipulation [42] or predetermined colour adjustments [43], such as randomly changing the brightness, contrast or colour properties of an image, see Figure 8.

Traditional photometric techniques in augmenting data may have limitations, e.g., high memory and computation requirements. In addition, they may result in crucial image information loss, particularly when the feature is a colorimetric feature capable of differentiating different dataset categories [44].

### 2.3. Kernel/Filter

Kernel plays an important role in deep learning. It can extract certain features from given images as a filter by sliding a window across the images. CNN models can learn features from images by automatically updating their kernel values according to the back-propagation process. Similarly, kernels with distinct values can also be used to conduct data augmentation and generate specific images containing specific features [10].

**Figure 9:**
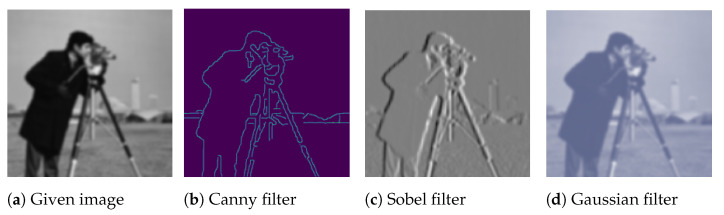
Data augmentation by using kernels/filters.

In computer vision, filters can be used for edge detection (e.g., using the Sobel [45] or Canny [46] filters), sharpening (e.g., using high-contrast vertical or horizontal edge filters), and blurring (e.g., using the Gaussian filter). In particular, edge enhancement that improves object edges within images can be used for data augmentation. It is hypothesised that using training images with augmented edges could improve CNN performance, since the learned kernels in CNN could detect objects’ shapes more easily [47]. Analogously, blurring images can also be utilised for data augmentation and could make models more resistant to blur or noise. Figure 9 shows an example of using different kernels/filers to augment images.

Using filters for data augmentation is a relatively unexplored field, even though the idea is straightforward. Its application in areas, such as action recognition, could be advantageous. For instance, edge detection filters may aid in recognising the human shape, thereby enabling the inference of its action. Motion blur may be used to augment data so as to improve models’ resistance to blurring in action recognition [48,49].

### 2.4. Noise Transformations

Noise is commonly defined as a random variation in brightness or colour information [50]. It is frequently caused by technical limitations of the image capture sensor or poor environmental conditions. Unfortunately, these issues are often unavoidable in actual situations, making image noise a prevalent problem to address.

**Figure 10:**
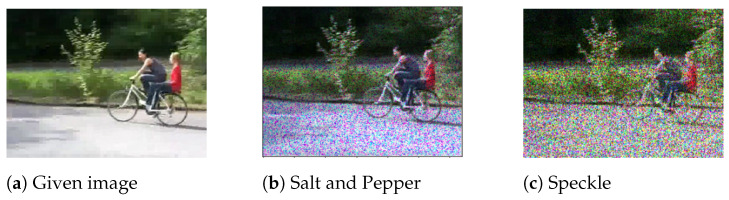
Data augmentation by using noise transformation.

Noise in data may appear to be a problem for neural networks in particular. Real-world data is rarely perfect [51]. When neural networks are evaluated on real-world data, noise can impair their accuracy and cause them to perform poorly in generalisation. At the very least, the data used to test deep learning models may not be as clean as the data used to train them. This may account for why deep neural network models frequently perform poorly in tests. Their robustness could be improved by augmenting data with different types of noise. Gaussian, salt and pepper, and speckle noise are three well-known forms of noise that can be used to augment image data [52], e.g., see Figure 10.

Gaussian noise is statistical noise with a probability density function equal to the normal distribution. The distribution of Gaussian noise is uniform throughout the signal [53]. Since it is additive noise, the pixels in a noisy image are made up of the sum of their original pixel values plus random Gaussian noise values. It is also independent at each pixel, and independent of the signal magnitude. Salt-and-pepper noise is also known as “spike noise” or “impulsive noise”. It causes white and black pixels to appear at random points in the image. This type of noise is mainly created by data transfer errors [54]. Speckle noise is multiplicative. It is generated by multiplying random values with different image pixels [53]. These different types of noise described above are generally dispersed over the image level. When they are used to augment data, deep learning models could be resistant to data that contains certain types of noise.

### 2.5. Random Erasing

**Figure 11:**
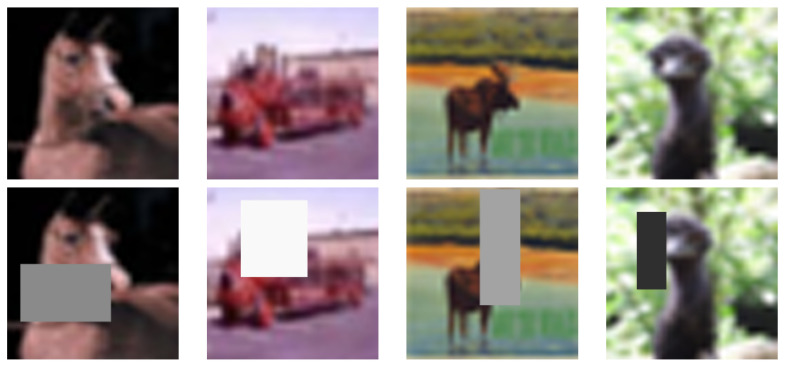
Data augmentation by the random erasing technique. The first and second rows represent the given images (from CIFAR-10) and the images after random erasing, respectively.

Random erasing [8] is a data augmentation technique which does not attempt to change individual image pixel values in general. Instead, it replaces the values of the pixels within a random size rectangle in an image by a random value, see Figure 11 for example. We could regard random erasing as a kind of noise technique focusing on local areas rather than individual pixels. It intends to make the model resistant to occlusion of objects in images (e.g., the datasets CIFAR-10, CIFAR-100, and ImageNet) and, thus, to reduce the possibility of overfitting. It enhances the data diversity holistically without increasing the data size, which is different from the other aforementioned data augmentation methods.

Since the random erasing technique selects a rectangular area (i.e., occlusion region) randomly, it may entirely erase the object information to be classified in the image. Therefore, it may not be recommended in categorising sensitive data which cannot withstand the deletion of a randomly generated local area in images, such as the cases of categorising licence plate numbers and letters.

## 3. Deep Learning-Based Data Augmentation Techniques

This section briefly recalls the most commonly used deep learning-based data augmentation approaches.

### 3.1. Texture Transfer

**Figure 12:**
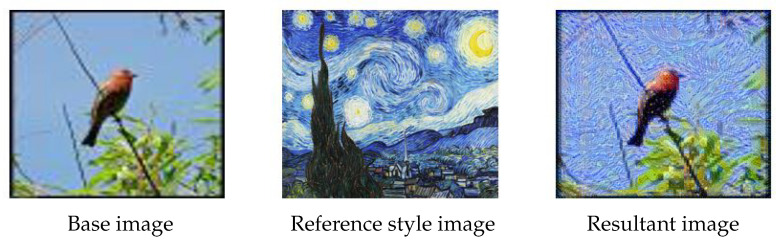
Data augmentation by texture transfer. Content of the base image (**left**) is mixed with the style of the reference style image (**middle**) to obtain the resultant image (**right**).

Texture transfer [55] aims to generate textures from source images while maintaining control over the semantic content of the source images, e.g., see Figure 12. It allows the generation of new images with given textures, while preserving the original images’ visual characteristics, such as contours, shading, lines, strokes and areas. The study in [56] demonstrated that CNNs are biased towards objects’ texture rather than shape, indicating that employing texture transfer may make a model more texture resistant.

The majority of traditional texture transfer methods resample textures into each particular content image [57]. For example, image quilting [55] creates a new image by stitching together small patches of other images. The work in [58] developed an image analogue technique, using pixel resampling to transfer textures from one image to another [59]. The newly generated images could be added into the training dataset to enlarge the data size and enhance its diversity.

### 3.2. Adversarial Training

**Figure 13:**
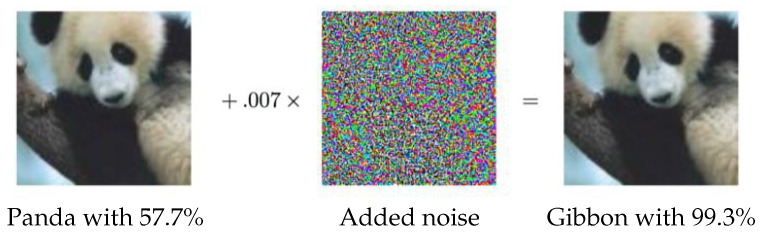
An adversarial example taken from [61]. Even though the given image and the image after adversarial noise added look exactly the same to the human eye, the noise fools the model successfully, i.e., the model labels the two images as different classes.

Adversarial examples, also known as machine illusion, have attracted considerable attention in the deep learning community. Adversarial examples can also be seen as members of the noise injection data augmentation family. By injecting a systematic noise into a given image, the CNN model outputs a completely different prediction, even though the human eye cannot detect the difference, see Figure 13. For example, the work in [60] created adversarial examples by changing a single pixel per image. Adversarial training is where these examples are added to the training set to make the model robust against attacks. As adversarial examples can detect weak points in a trained model, this way of augmenting data can be seen as an effective data augmentation approach.

### 3.3. Generative Adversarial Networks for Data Augmentation

Inspired by adversarial examples, the generative adversarial network (GAN), proposed in [62], has been widely used for data augmentation. Synthetic images created by GANs, which even humans find difficult to distinguish from the real images, help models significantly increase their robustness. GAN consists of two networks, i.e., a generator, which creates new images, and a discriminator, which tries to detect if the generated images are real or fake. For the variants of GANs, please refer to e.g., DCGAN [63], progressively growing GANs [64] and CycleGANs [65].

## 4. Data Augmentation in Image Classification and Segmentation

Data augmentations performed using traditional transformation techniques is still the most popular among academics, due to their simplicity [10,66]. Often, traditional and deep learning-based augmentation approaches are used either separately or in tandem. Image classification and image segmentation are two common, yet important, research areas in computer vision, which typically use data augmentation approaches. In this section, we discuss recent research, mostly within the past five years, in these two areas that leverage data augmentation for performance enhancement.

### 4.1. Data Augmentation on Image Classification

Lots of works have used data augmentation in image classification tasks, and their results vary, depending on aspects such as, models, data and applications. See Table 1 for a brief survey in this respect.

In 2017, the work in [67] suggested that deep learning-based augmentation methods, like GANs, do not perform significantly better than traditional techniques, but consume nearly three times more computational cost. Moreover, in [67], a model called “SmallNet” was trained using traditional augmentation techniques and style transfer with CycleGAN [81]. It was observed that combining deep learning-based methods with traditional techniques could achieve better results. Hussain et al. [68] evaluated various augmentation strategies on a medical image dataset using VGG-16. They demonstrated that the flipping and Gaussian filter augmentation techniques yielded superior outcomes compared to the other ones, particularly when adding noise, which gave the lowest accuracy. Pawara et al. [69] applied data augmentation techniques, such as rotation, blur, contrast, scaling, illumination, projective transformation, and multiple combinations of these techniques to enhance plant image classification performance. In this challenge, pre-trained and untrained AlexNet and GoogleNet models were used. It was observed that CNN models trained from scratch benefited significantly from data augmentation, whereas pre-trained CNN models did not. In addition, it was discovered that combinations of data augmentation techniques like rotation and varied illuminations could contribute most for CNN models trained from scratch in achieving excellent performance.

In 2018, Inoue et al. [70] developed a new technique, known as SamplePairing, in which a new sample was synthesised from one image by overlaying another image randomly selected from the training data, i.e., taking an average of two images. Li et al. [71] found that traditional data augmentation techniques were not cumulative, and that a threefold increase in sample size was often sufficient to reach the upper bound. In addition, the PBP technique proposed by the authors significantly increased the number of samples, and was proved to be effective for hyperspectral imagery classification. FridAdar et al. [72] classified liver lesions using a small customised CNN architecture. In order to accommodate small datasets and input sizes, they suggested that CNN designs should often contain fewer convolutional layers. By combining traditional data augmentation techniques with GAN-based synthetic images, more accurate results from a small dataset were obtained. Pham et al. [73] discussed how to solve the challenges of skin lesion classification and limited data in medical images by applying image data augmentation techniques, such as geometric augmentation and colour augmentation. The effects of a different number of augmented samples were evaluated on the performance of different classifiers, and it was concluded that the performance of skin cancer classifiers and medical image classifiers could be improved by utilising data augmentation. Motlagh et al. [74] classified several forms of cancer using 6402 tissue microarrays (TMAs) as training samples and utilising transfer learning and deep neural networks. Data augmentation techniques, such as random scaling, rotation, cropping, and flipping, were used to obtain sufficiently different samples, and the results showed that 99.8 percent of the four cancer types, including breast, bladder, lung and lymphoma, were correctly classified using the ResNet50 pre-trained model.

In 2019, Zheng et al. [75] assessed the efficacy of neural style transfer using VGG16 on the Caltech 101 and Caltech 256 datasets, and the results demonstrated a two-percent gain in accuracy. Recent research has demonstrated that neural style transfer algorithms can apply the artistic style of one image to another image without altering the latter’s high-level semantic content, showing that neural style transfer can be used for data augmentation to add more variation to the training dataset.

In 2020, Ismael et al. [76] employed data augmentation to solve the problem of insufficient training data and imbalanced classes in the MRI image classification task for brain cancer. Various augmentation techniques, including horizontal and vertical flipping, rotating, shifting, zooming, shearing and brightness alteration, were utilised. They observed that each augmentation technique had different effects on the performance of distinct classes. For instance, manipulation of brightness yielded 96 percent accuracy for class one, whereas the rotation technique yielded 98 percent accuracy for the same class. For class two, these two techniques achieved a score of 99 percent with brightness and 98 percent with rotation. By combining all of the previously mentioned augmentation techniques, they were able to attain 99 percent overall accuracy, i.e., 4 percent improvement against the results obtained without data augmentation. Additionally, Gour et al. [77] developed ResHist, a 152-layer CNN based on residual learning, for breast cancer histopathological image classification. A data augmentation strategy was devised, based on stain normalisation, image patch generation and affine transformation, to improve the model performance. Experimental results demonstrated that with the help of data augmentation the model performance for classifying histopathology images was better than the pre-trained networks, including AlexNet, VGG16, VGG19, GoogleNet, Inception-v3, ResNet50 and ResNet152.

In 2021, Kandel et al. [80] examined the impact of test time augmentation (TTA) on X-ray images for bone fracture detection using the MURA dataset. It was observed that TTA could dramatically improve classification performance, especially for models with a low score, by comparing the performance of nine different augmentation techniques with five state-of-the-art CNN models. Nanni et al. [78] investigated the performance of over ten different kinds of data augmentation techniques, including kernel filters, colour space transforms, geometric transformations, random erasing/cutting and image mixing, and proposed two approaches: the discrete wavelet transform and the constant-Q Gbor transform. Using the aforementioned data augmentation techniques, the performance of several ResNet50 networks was evaluated on four benchmark image datasets (i.e., a virus dataset, a bark dataset, a portrait dataset, and a LIGO glitches dataset), representing diverse problems and different scales, indicating the efficacy of data augmentation techniques in enhancing model performance. In addition, the work in [79] investigated the impact of augmenting ECG images for COVID-19 and cardiac disease classification using deep learning. They argued that traditional data augmentation did not improve the performance of neural networks in their experiments with ECG signal images.

### 4.2. Data Augmentation on Image Segmentation

Image segmentation is also an important field in computer vision. It involves grouping an image into different parts where each part may share certain features and characteristics. It has a close relationship to image classification. For example, image segmentation, in some sense, could be achieved by classifying individual pixels in an image into different groups. A great deal of emphasis has been placed on data augmentation in order to achieve better segmentation results, particularly when working with small training datasets. For practical semantic segmentation applications, collecting and annotating sufficient training data for deep neural networks is notoriously difficult. Therefore, data augmentation techniques are of great importance. We, below, survey a number of studies that have involved data augmentation in image segmentation tasks. See Table 2 for a summary of relevant literature.

In 2018, the work in [82] used an encoder–decoder structure, adapted from an hourglass network, prevalent in the field of human-pose estimation [83], in order to classify and segment brain tumours in MRI scans for the BraTS 2018 challenge [84,85,86,87]. Two data augmentation techniques were utilised: vertical flipping, which matches up to the naturally symmetrical shape of the brain, and random intensity variation, used because the intensity between MRI scans varies significantly. The network was trained with, and without, data augmentation. It was discovered that data augmentation appeared to provide a small increase in accuracy for the Dice coefficient and a significant improvement in Hausdorff accuracy.

**Table 2:** Survey of data augmentation techniques in recent image segmentation works.

Papers	Dataset	Aug. Techniques	Model	Task	Findings
Benson et al., 2018 [82]	BraTS 2018	Vertical flipping and random intensity variation.	Hourglass Network [83]	Brain tumor segmentation	Data augmentation methods appear to have a different impact on the Dice coefficient and Hausdorff accuracy.
Casado et al., 2019 [88]	ISBI challenge [89].	Automated image augmentation tool.	U-Net architecture with four different models	Semantic segmentation	Introduced an approach that enables researchers to use image augmentation techniques automatically to the challenges of object classification, localisation, detection, semantic segmentation, and instance segmentation.
Ma et al., 2019 [90]	Sheep segmentation dataset (SSG)	Colour transformation, flipping, cropping, projection transformation, local copy, and JPEG compression.	DeepLabv3+ [91]	Semantic segmentation	A combination of augmentation methods could achieve the best performance, while excessive augmentation could degrade the performance.
Qiao et al., 2020 [92]	Cattle segmentation dataset	Random image cropping and patching.	Bonnet [93]	Semantic segmentation	The proposed method of randomly cropping and patching images to increase the number of training images improves segmentation performance.
khryashchev et al., 2020 [94]	Planet, and the Resurs datasets	Random chromatic distortion, rotation, and shifting.	U-Net with the ResNet34 as encoder	Semantic segmentation	The application of random chromatic distortion in HSV colour format improves the robustness of deep learning algorithms for images with noise, such as small clouds and glare from reflective surfaces.
Chen et al., 2020 [95]	Tongue image dataset	Cropping, rotation, flipping, and colour transformations.	U-Net with 15 different CNN models as encoders	Semantic segmentation	Geometric transformations can achieve higher performance than colour transformations, and segmentation accuracy can be increased by 5 to 20% compared to no augmentation.
Qin et al., 2020 [96]	Kidney Tumour dataset	An automatic deep reinforcement learning based augmentation method	An end-to-end augmentation segmentation architecture	Medical image segmentation	Conventional augmentation techniques (e.g., rotation, cropping, etc.) are random and sometimes damaging to the image segmentation task.
Cirillo et al., 2021 [97]	BraTS2020 Dataset	Flipping, rotation, scaling, brightness adjustment, and elastic deformation.	3D U-Net [98]	3D brain tumor segmentation	Conventional data augmentation significantly improves the validation performance of brain tumour segmentation.
Su et al., 2021 [99]	Narrabri and Bonn datasets	Random image cropping and patching (RICAP) method.	Bonnet	Semantic segmentation	The RICAP technique increases the mean accuracy and mean intersection over union (IOU) of the CNNs with the traditional data augmentation.
Zhang et al., 2021 [100]	PASCAL VOC 2012; Cityscapes; CRAG dataset	Object-level augmentation method.	MobileNet based DeepLab V3+	Semantic segmentation	ObjectAug can easily be integrated with existing image-level augmentation techniques to further improve the segmentation performance. ObjectAug supports category-aware augmentation that gives objects in each category a variety of options.
Mallios et al., 2021 [101]	VoxTox [102]	GAN-based synthetic images.	RS-FCN	Rectum segmentation	Demonstrated the viability of producing synthetic data and subsequently incorporating it into the training samples in order to get satisfactory outcomes.

In 2019, Casado et al. [88] presented a versatile method which was implemented in the open-source package CLoDSA, dedicated to classification, semantic segmentation, instance segmentation, localisation and detection. Three different datasets were used to demonstrate the benefits of applying data augmentation. Ma et al. [90] created the SSG dataset, i.e., a small-scale and open-source sheep segmentation dataset containing hundreds of images. To find the best technique for this small semantic segmentation dataset, they evaluated seven data augmentation methods, including colour transformation, flipping, cropping, projection transformation, local copy, a proposed technique named “JPEG compression” and their combinations. Experimental results showed that the combination of compression, cropping and local shift could achieve the best augmentation performance for their AI-Ranch application. However, they also found that excessive augmentation could degrade performance.

In 2020, Qiao et al. [92] introduced a data augmentation technique where images were randomly cropped into distinct regions and then patched together to form a new one. Experimental results on their acquired cattle dataset showed that this data augmentation technique, together with an open-source semantic segmentation CNN architecture, “Bonnet” [93], achieved 99.5 percent mean accuracy and 97.3 percent mean intersection of unions. In [94], an U-Net neural network with the ResNet34 encoder was used for automated wildfire detection on high-resolution aerial photos using two small satellite RGB image datasets. To overcome the small data size challenge, data augmentation techniques, such as rotation, shifting and random chromatic distortion in HSV colour format, were used to increase the robustness of the deep learning algorithm for noisy images, such as small clouds and glare from reflective surfaces. The experimental results showed that data augmentation methods led to better results on test datasets for all metrics used in the experiments. Qin et al. [96] argued that the data generated by conventional augmentation techniques (e.g., rotation, cropping, etc.) was random and sometimes detrimental to the image segmentation process. In light of this, an automatic learning-based data augmentation technique was developed for CT kidney tumor segmentation.

The work in [95] focused on automatic tongue segmentation using 15 different pre-trained network models (such as VGG, ResNet, ResNext, DenseNet, EfficientNet, inceptionV3, SE-ResNet, inception, ResNetV2, etc.). They utilised multiple label-preserving transformations to increase the size and diversity of the training dataset. Their findings indicated that geometric transformations could achieve greater performance than colour transformations, and that the segmentation accuracy could be improved by 5 to 20 percent compared to no augmentation.

In 2021, the work in [100] proposed a data augmentation technique, named ObjectAug, for image segmentation. The ObjectAug technique operates at the object level by first decoupling the image into individual objects and the background using semantic labelling and, then, each object is individually augmented using conventional augmentation techniques (e.g., scaling, shifting and rotation), followed by image inpainting, which is utilised to further restore the pixel artefacts introduced by object augmentation. The final step is integrating the augmented objects and background into an augmented image. Extensive experiments on both normal and medical image datasets demonstrated that the ObjectAug technique outperformed conventional augmentation techniques and improved segmentation performance. Cirillo et al. [97] examined how augmentation techniques, such as flipping, rotation, scaling, brightness adjustment and elastic deformation, affected the learning process when training a standard 3D U-Net [98] on the BraTS dataset [85,103,104]. In multiple cases, their findings indicated that data augmentation significantly improved validation performance. They presumed that the reason why data augmentation had not been thoroughly investigated for brain tumour segmentation was because the BraTS training set was quite large and several works [105,106] suggested that data augmentation would not be of much assistance.

Mallios et al. [101] investigated image-guided radiation therapy [102,107], which is one of the most prevalent methods for treating numerous types of cancer. Their study included the development of deep learning approaches for segmenting the organs-at-risk in CT images during radiation therapy. It was observed that the scarcity of annotated data, stemming from the difficulty and time consuming nature of manual annotation in this area, hindered research development for medical applications. In order to compensate for the shortage of labelled real-world data required to train very deep models, like FCN architecture [108], cGAN [109] was used to generate synthetic images. The experimental results illustrated the superior performance of the proposed segmentation methods for the rectum under the help of deep learning-based data augmentation. In [99], a framework for augmenting data for semantic segmentation, based on the random image cropping and patching (RICAP) method, was presented. Experiments on two datasets using Bonnet architecture [93] showed that the developed framework improved segmentation performance in terms of accuracy and mean intersection over union.

## 5. Proposed Strategy for Data Augmentation

In this section, we propose a new data augmentation technique, belonging to the traditional data augmentation category. It was inspired by techniques focusing on local areas in images, e.g., the random erasing technique.

Let D be the training dataset. Let $C_{x,y,r}$ be a circular area in an image I∈D, with centre location (x,y) and radius *r*. Let θ∈[0,2π] be an angle for rotation.

**Figure 14:**
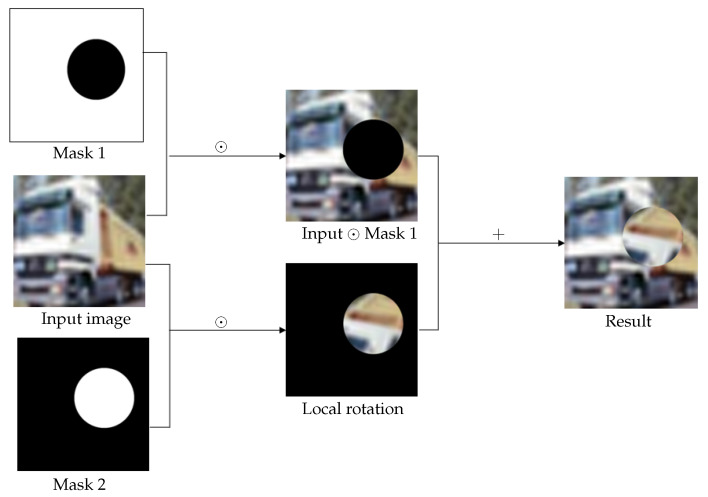
The proposed random local rotation data augmentation strategy. Symbol ⊙ represents pointwise multiplication.

The main procedure of the proposed augmentation technique is given below. Firstly, ∀I∈D, we select a circular area $C_{x,y,r}$ within image ***I***, with a randomly generated centre (x,y) and radius *r*. Then, the image content within the circular area $C_{x,y,r}$ is rotated with a randomly generated angle θ∈[0,2π], while the image content outside the circular area $C_{x,y,r}$ is kept, and we call this newly generated image $\tilde{I}$. Finally, image $\tilde{I}$ is used to augment the original training dateset D. Here we suggest two ways. The first one is to use the generated image $\tilde{I}$ to replace the original image I∈D. This way does not change the size of the dataset D, but may change the data diversity. The other way is to add image $\tilde{I}$ into the dataset D, which increases the dataset size and enhances the data diversity. We call the above technique random local rotation (RLR), see Figure 14 for the diagram showing the conducting of the RLR data augmentation strategy and Algorithm 1 for the summary of RLR.
**Algorithm 1** Random Local Rotation 1:**Input:** The training dataset D 2:**Output:** The augmented training dataset $\tilde{D}$ 3:Create a subset say $D^{\prime }\subseteq D$ by randomly selecting *N* images from D 4:Create an empty set say $D^{*}$ 5:**for**$\forall I\in D^{\prime }$**do** 6:    Randomly generate centre (x,y) and radius *r* within image ***I*** 7:    Form a circular area $C_{x,y,r}$ within image ***I*** with centre (x,y) and radius *r* 8:    Randomly generate angle θ∈[0,2π] 9:    Form image $\tilde{I}$ by rotating the area within $C_{x,y,r}$ in image ***I*** with angle θ10:   Add $\tilde{I}$ into $D^{*}$11:**end for**12:Way 1: $\tilde{D}\leftarrow D^{*}\cup D\setminus D^{\prime }$13:Way 2: $\tilde{D}\leftarrow D^{*}\cup D$

**Figure 15:**
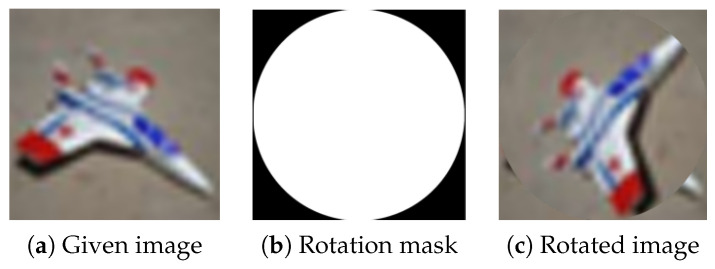
Random local rotation data augmentation technique using the largest possible circular rotation area in the image centre.

A special case of RLR uses the largest possible circular rotation area in the image centre, see Figure 15. In the rest of this article, we call this special case random centre rotation (RCR). RCR could be applied as a direct replacement of the traditional rotation technique for data augmentation.

An obvious advantage of RLR against the traditional rotation is that it avoids the black boundary caused by traditional rotation, as shown in Figure 4. Moreover, the local area information distortion brought by RLR could improve the data diversity, without removing much information from the given images, like other augmentation techniques, e.g., image cropping, random erasing, etc. Detailed validation of RLR is presented in the next section.

## 6. Experiments

To validate the proposed RLR agumentation technique, we employed three state-of-the-art CNN models, i.e., ResNet50 [22], MobileNet [110] and InceptionV3 [23], which were all trained from scratch. We conducted experiments in both classification and segmentation tasks and mainly compared with the traditional rotation technique (shortened to TR) with randomly selected rotation angles. The quantitative results reported below with standard deviation were obtained by repeating the experiments five times.

### 6.1. Classification Experiment

The CIFAR-10 dataset was selected for conducting experiments regarding the classification task. It contained 60,000 coloured images, where every image was of size 32×32, A total of 50,000 images were for training and 10,000 images were for testing. CIFAR-10 consisted of ten classes, each with 6000 images. To simulate the scenario of data scarcity, we reduced the original training data size to 2%, 4% and 6%, forming three subsets with numbers of samples of 1000, 2000 and 3000, respectively, and used the original test set for testing.

For each subset, three extra copies were created by the TR, RCR and RLR data augmentation techniques. Each augmented copy was twice as large as its corresponding original subset. The data balance between the classes was also taken into consideration when constructing these subsets. Additionally, the image resolution was adjusted to fit the default input shape of each CNN model used in the experiments, i.e., 299×299 for InceptionV3, and 244×244 for MobileNet and ResNet50. According to the constructed datasets, each model was subjected to a total of 12 tests, (i.e., number of subsets × number of techniques).

For fair comparison, same hyperparameters were kept for each model. Models were trained for 50 epochs with the Adam optimiser and categorical cross entropy loss function. Test accuracy was selected as the monitoring metric. The Spyder platform was utilised to train and evaluate the models.

#### 6.1.1. Classification Results

Table 3 gives the classification accuracy of the CNN models (i.e., ResNet50, MobileNet and InceptionV3) with the TR, RCR and RLR data augmentation techniques on the three subsets, including the comparison with the baseline results (i.e., the ones obtained on the subsets without using data augmentation).

The results in Table 3 show that our proposed data augmentation technique RLR constantly achieved the best classification accuracy with all the three CNN models on all the subsets, indicating its excellent performance. In contrast, the traditional rotation technique did not improve performance, and, in many cases, degraded the results, compared with the baseline results. This might be because of the aforementioned limitations of the traditional rotation technique, i.e., the black and irregular boundary it introduces. As for the performance of the RCR technique, its results were slightly better than the TR results and were comparable to the baseline results. This was what we expected, since RCR is quite similar to TR. Yet, the images generated by RCR did not suffer from the black and irregular boundaries, and, therefore, performed slightly better than TR.

For further performance evaluation, we also reported the comparison of the RLR method with the mostly used traditional data augmentation techniques, see Table 4. The smallest subset of CIFAR-10 (i.e., the one with 1000 samples) was employed with data augmentation techniques, including RLR, RNR, RWR, RRR, flipping, shifting, zooming, and brightness. The results in Table 4 show that, generally, data augmentation techniques could indeed enhance the performance of different models. It again demonstrated the great performance of the proposed RLR method; for example, RLR achieved the best accuracy when the ResNet model was used. The results in Table 4 also show that the performance of the augmentation techniques might differ for different models, which is worth investigating further in the future.

#### 6.1.2. Qualitative Comparison via Saliency Maps

To further evaluate the effectiveness of the proposed RLR technique against the traditional rotation, we employed GradCAM [111], one of the well-known methods illustrating the decision made by CNNs, to show the saliency maps regarding the TR and RLR techniques.

**Figure 16:**
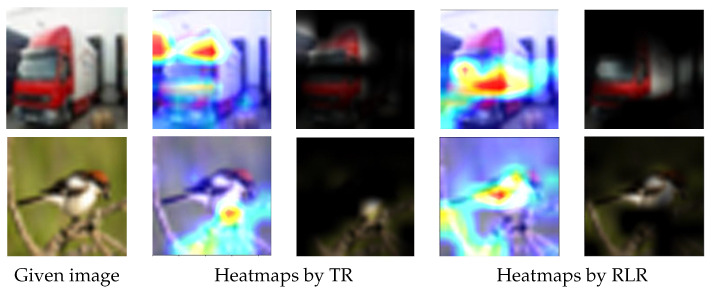
Data augmentation techniques evaluation by saliency map. Column 1: given images; columns 2 and 3: two types of saliency maps for TR; columns 4 and 5: two types of saliency maps for RLR. In particular, for the two types of saliency maps evaluating each data augmentation technique, the first saliency map highlights the activated area in the given image, and the second highlights the activated area using the content of the given image. The CNNs used for the test images in the first and second rows are MobileNet and ResNet50, respectively. The saliency maps created for the models, which were trained with the dataset augmented with RLR, clearly focus on the wider part of the object while for the other cases where augmentation is achieved with TR, the models focus on a smaller area of the object. The models trained with RLR output more reliable results, together with the wider focus on the target object shown in the above saliency maps, demonstrating the superior performance of RLR compared to TR.

Figure 16 shows the saliency maps of the TR and RLR techniques on images randomly selected from the test datasets. The truck image (first row in Figure 16) was classified as truck with 94% (here the percentage was the probability produced by the Softmax activation function in the CNN architectures) via MobileNet model trained with the TR augmentation technique, and nearly 100% with the proposed RLR technique. The bird image (second row in Figure 16) was classified as bird with 95% via ResNet50 model trained with the TR augmentation technique, and nearly 100% with the proposed RLR technique. The saliency maps shown in Figure 16 for the TR and RLR techniques indicated that the proposed RLR technique was, indeed, more effective in terms of assisting the CNN architectures to make decisions based on more reasonable areas within the test images.

### 6.2. Segmentation Experiment

**Figure 17:**
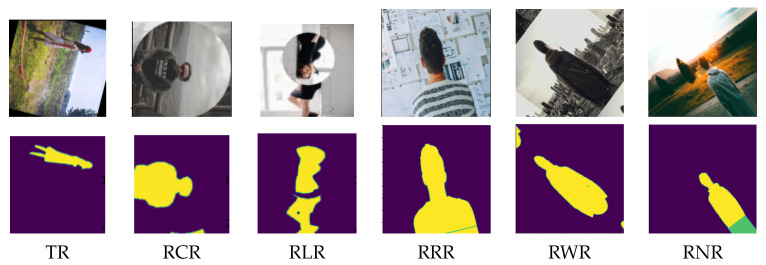
Samples of the Supervisely Person dataset by applying the RLR, TR, RCR, RRR, RWR and RNR augmentation techniques. Rows one and two are the augmented samples with their corresponding human body segmentation, respectively.

**Figure 18:**
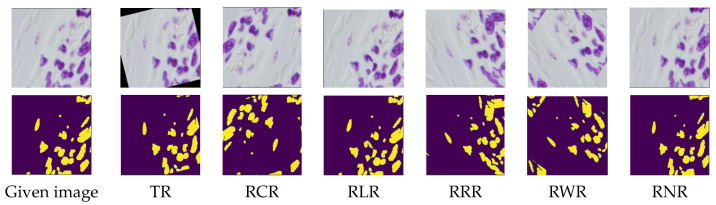
Samples of the Nuclei images dataset by applying the RLR, TR, RCR, RRR, RWR and RNR augmentation techniques. Rows one and two are the augmented samples with their corresponding segmentation, respectively.

Two publicly available datasets were selected for conducting experiments regarding the segmentation task. The first dataset was the Supervisely Person [112], which contained 5711 images and 6884 high-quality annotated human instances for human semantic segmentation, see e.g., Figure 17. The second dataset was the Nuclei images dataset [113], which contained 670 microscopic images with their corresponding segmentation masks, see e.g., Figure 18. Each augmented copy was twice as large as its corresponding original dataset. Then, each dataset copy was divided into training (90% of the data) and validation (10% of the data) subsets. Note that, in this experiment, we also considered the concept of equivariance. Equivariance implies that the output changes in proportion to the input. The concept of equivariance is important in segmentation, where the location of the object and the location of the segmented object shift proportionally, e.g., see Figure 17 and Figure 18. In contrast, invariance refers to a change in the location of an object while the output remains unchanged, which is considered in Section 6.1 for the classification task.

Two autoencoders were used to conduct the semantic segmentation task. These two autoencoders were constructed based on two models (i.e., MobileNet and VGG16), each with a customised decoder, see Table 5 for the detailed architectures. Each autoencoder was subjected to a total of seven tests. They were trained for 200 epochs with the Adam optimiser and binary cross entropy loss function.

#### Segmentation Results

Table 6 gives the segmentation accuracy of the autoencoders (i.e., MobileNet-based, and VGG16-based) with the TR, RCR, RLR, RNR, RWR and RRR data augmentation techniques on the Supervisely Person dataset, including the comparison with the baseline results (i.e., the one obtained on the original samples without using data augmentation). In contrast to the classification results, the results in Table 6 show that all the augmentation techniques tested did not improve the segmentation performance. This might imply that using rotation alone to augment data might not be a good method for the segmentation task, particularly if the shape feature was the most important one in the dataset, as in the Supervisely Person dataset. For further investigation into the influence of different features on the performance of the augmentation techniques, we conducted an experiment using the Nuclei images dataset [113].

**Figure 19:**
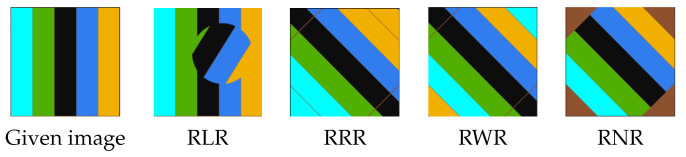
The effect of different rotation methods on the rotated image. The RRR and RWR expanded the central region (i.e., the black stripe) by repeating parts of it. RNR resulted in the loss of image content at the image’s periphery and the creation of artificial pixel values to fill the gap. In contrast, RLR manipulated the content of the image while preserving the information around the image’s periphery well.

Differing from the results obtained in Table 6 on the Supervisely Person dataset, the results in Table 7 on the Nuclei images dataset demonstrated that the rotation augmentation methods could improve the segmentation performance. This performance gain might be due to the fact that the augmentation techniques did not degrade the image qualities much in the Nuclei dataset, since the shape feature was not that critical, compared to the Supervisely Person dataset. In the Nuclei Images dataset, colours and textures are likely to be more essential than the shape features. In particular, the segmentation results in Table 7 on the Nuclei dataset showed that RLR achieved the best performance among the rotation augmentation methods. This might be due to the information preservation ability that RLR provided, whereas RRR, RWR and RNR either lost parts of the information in the image’s periphery or repeated some parts of the image, see Figure 19.

### 6.3. Discussion

The vast majority of researchers combine many data augmentation techniques to obtain a final result. This makes it difficult to acquire an accurate evaluation for these techniques individually. In this study, we chose the random rotation technique and examined it in more detail, along with its impact on two significant tasks (i.e., classification and segmentation), in order to make a contribution to the data augmentation regime in general. Segmentation and classification are two distinct tasks. The notion that both rely on the same features to attain their desired outcomes may not be accurate. Our results in the previous section showed that the rotation augmentation techniques could enhance methods’ performance for the classification task, but not the segmentation task. It was observed that the segmentation task naturally relied on shape features [114]. Geirhos et al. [56] conducted a quantitative experiment demonstrating that CNNs trained with ImageNet had a strong inclination to classify texture over shape. This feature distinction might account for the disparity between classification and segmentation results when the rotation augmentation techniques were applied. In particular, in the segmentation experiment, the RLR method distorted the shape of the human body the most, yielding a slightly poorer result than that of the TR method, which did not distort the shape of the human body. The distortion of the shape feature might explain the deterioration of the segmentation results when applying the rotation augmentation techniques. In contrast, for the classification task, the rotation augmentation techniques altered the object shape but not the overall texture, which benefited the performance enhancement for the classification task.

## 7. Conclusions

Deep learning models, like CNNs, are susceptible to overfitting. In this work we surveyed the data augmentation techniques, particularly recent research in image classification and segmentation employing data augmentation techniques, which are critical for deep learning models to overcome the overfitting issue and achieve better performance. In addition, we proposed a geometric augmentation technique, i.e., RLR (random local rotation), focusing on manipulating local information within images without adding non-original pixel values. Quantitative and qualitative experimental results demonstrated that RLR could be more effective than the traditional rotation technique in classification and some segmentation tasks, and, therefore, complemented the existing data augmentation techniques well.

## Figures and Tables

**Table 1:** Survey of data augmentation techniques in recent image classification works.

Papers	Dataset	Aug. Techniques	Model	Task	Findings
Shijie et al., 2017 [34]	CIFAR10; ImageNet (10 categories)	GAN/WGAN, flipping, cropping, shifting, PCA jittering, colour jittering, noise, rotation.	AlexNet	Image classification	Four methods (i.e., cropping, flipping, WGAN and rotation) perform generally better than other augmentation methods, and some appropriate combination methods are slightly more effective than the individuals.
Perez et al., 2017 [67]	A small subset of ImageNet; MNIST.	Neural augmentation, CycleGAN, GANs, cropping, rotating, and flipping.	SmallNet	Image classification	GANs do not perform better than traditional techniques.
Hussain et al., 2017 [68]	Digital Database for Screening Mammography (DDSM).	Flipping, cropping, noise, Gaussian filters, principal component analysis (PCA).	VGG-16	Medical images classification	The flipping and Gaussian filter techniques are better than noise transformation.
Pawara et al., 2017 [69]	Folio, AgrilPlant, and the Swedish Leaf datasets	Rotation, blur, contrast, scaling, illumination, and projective transformation.	AlexNet; GoogleNet	Plant image classification	CNN models trained from scratch benefit significantly from data augmentation.
Inoue et al., 2018 [70]	ILSVRC2012; CIFAR-10	SamplePairing, flipping, distorting, noise, and cropping.	GoogLeNet	Image classification	Developed a new technique known as SamplePairing.
Li et al., 2018 [71]	Indian Pines and Salinas datasets	Pixel-block pair, flipping, rotation, and noise.	PBP-CNN	Hyperspectral imagery classification	A threefold increase in sample size is often sufficient to reach the upper bound.
Frid-Adar et al., 2018 [72]	Liver lesions dataset	Translation, rotation, scaling, flipping and shearing, and GAN-based synthetic images.	Customised small CNN architecture	Medical image classification	Combining traditional data augmentation with GAN-based synthetic images improves small datasets.
Pham et al., 2018 [73]	Skin lesion dataset (ISBI Challenge)	Geometric augmentation and colour augmentation.	InceptionV4	Skin cancer image classification	Skin cancer and medical image classifiers could benefit from data augmentation.
Motlagh et al., 2018 [74]	Tissue Micro Array; Breast Cancer Histopathological Images (BreaKHis)	Random resizing, rotating, cropping, and flipping.	ResNet50	Breast cancer image classification	Traditional data augmentation techniques are adequate for obtaining distinct samples of various types of cancer.
Zheng et al., 2019 [75]	Caltech 101; Caltech 256.	Neural style transfer, rotation, and flipping.	VGG16	Image classification	Neural style transfer can be utilised as a deep-learning data augmentation technique.
Ismael et al., 2020 [76]	Brain tumor dataset	Horizontal and vertical flips, rotating, shifting, zooming, shearing, and brightness alteration.	ResNet	MRI image classification (Brain Cancer)	The effectiveness of traditional augmentation methods varied among classes.
Gour et al., 2020 [77]	BreaKHis dataset	Stain normalisation, image patch generation, and affine transformation.	ResHist model	Breast cancer histopathological image classification	The model performance for classifying histopathology images is better with data augmentation than with pre-trained networks.
Nanni et al., 2021 [78]	Virus, a bark, a portrait, and a LIGO glitches datasets	Kernel filters, colour space transforms, geometric transformations, and random erasing.	ResNet50	Image classification	Introduced the discrete wavelet transform and the constant-Q Gbor transform as two new methods for data augmentation.
Anwar et al., 2021 [79]	Customised image based ECG signals	Flipping, cropping, contrast and Gamma distortion.	EfficientNet B3	ECG images classification	In the experiment with images of ECG signal, traditional data augmentation did not improve the performance of neural networks.
Kandel et al., 2021 [80]	MURA dataset	Horizontal flip, vertical flip, rotation, and zooming.	VGG19; ResNet50; InceptionV3; Xception; DenseNet121	X-ray images classification	Augmentation was found to significantly enhance classification performance.

**Table 3:** Classification accuracy comparison between the TR, RCR and RLR data augmentation techniques. CNN models, i.e., MobileNet, ResNet and InceptionV3, with the data augmentation techniques, were applied on three different CIFAR-10 subsets, with numbers of samples of 1000, 2000 and 3000, respectively. The results indicated the superior performance of the proposed RLR technique.

Model	Subset	Baseline	RLR	TR	RCR
	1000	41.69±0.29	42.24±0.44	40.57±0.22	39.51±0.52
MobileNet	2000	50.62±0.43	51.76±0.56	48.77±0.51	50.6±0.64
	3000	56.95±0.62	60.96±0.54	55.30±0.82	58.18±0.66
	1000	39.73±0.64	41.47±0.39	38.11±0.52	38.28±0.51
ResNet	2000	50.16±0.49	51.06±0.54	47.95±0.84	48.84±0.59
	3000	53.78±0.49	56.15±0.76	53.38±0.56	53.31±0.56
	1000	42.65±0.63	45.41±0.55	43.32±0.47	43.15±0.58
InceptionV3	2000	54.63±0.45	55.71±0.48	54.85±0.57	53.78±0.28
	3000	61.24±0.37	62.45±0.86	59.72±0.42	60.18±0.66

**Table 4:** Classification accuracy comparison between RLR and other common data augmentation techniques. CNN models, i.e., MobileNet, ResNet and InceptionV3, with the data augmentation techniques, were applied on the smallest subset of CIFAR-10 (i.e., the one with 1000 samples).

Model	Baseline	RLR	RNR	Flip	Shift	Zoom	Bright	RWR	RRR
MobileNet	41.89	42.28	41.37	42.52	45.83	45.75	47.84	45.53	45.10
ResNet	39.40	41.70	40.07	40.18	41.57	40.19	39.06	41.22	41.18
InceptionV3	42.85	45.61	44.84	45.86	43.03	45.81	46.11	46.67	45.41

**Table 5:** The architectures of the decoders of the MobileNet-based and VGG16-based auto-encoders. Conv2D and Conv2DT represent the 2D convolutional layer and the transposed 2D convolutional layer, respectively.

MobileNet Decoder				VGG16 Decoder			
**Layer**	**Kernel**	**Filters**	**Activation**	**Layer**	**Kernel**	**Filters**	**Activation**
	**Size**	**Number**	**Function**		**Size**	**Number**	**Function**
Conv2DT	(3,3)	1024	Relu	Conv2DT	(3,3)	1024	Relu
Batch Normalisation				Batch Normalisation			
Conv2D	(3,3)	1024	Relu	Conv2D	(3,3)	1024	Relu
Batch Normalisation				Batch Normalisation			
Conv2DT	(3,3)	512	Relu	Conv2DT	(3,3)	512	Relu
Batch Normalisation				Batch Normalisation			
Conv2D	(3,3)	512	Relu	Conv2D	(3,3)	512	Relu
Batch Normalisation				Batch Normalisation			
Conv2DT	(3,3)	256	Relu	Conv2DT	(3,3)	256	Relu
Batch Normalisation				Batch Normalisation			
Conv2D	(3,3)	256	Relu	Conv2D	(3,3)	256	Relu
Batch Normalisation				Batch Normalisation			
Conv2DT	(3,3)	128	Relu	Conv2DT	(3,3)	128	Relu
Batch Normalisation				Batch Normalisation			
Conv2D	(3,3)	128	Relu	Conv2D	(3,3)	128	Relu
Batch Normalisation				Batch Normalisation			
Conv2DT	(3,3)	64	Relu	Conv2DT	(3,3)	64	Relu
Batch Normalisation				Batch Normalisation			
Conv2D	(3,3)	64	Relu	Conv2D	(3,3)	64	Relu
Batch Normalisation				Batch Normalisation			
Conv2DT	(3,3)	32	Relu	Conv2DT	(3,3)	32	Relu
Batch Normalisation				Batch Normalisation			
Conv2D	(3,3)	32	Relu	Conv2D	(2,2)	32	Relu
Batch Normalisation				Batch Normalisation			
Conv2D	(3,3)	1	Sigmoid	Conv2D	(3,3)	1	Sigmoid

**Table 6:** Segmentation accuracy comparison between different data augmentation techniques (i.e., TR, RCR, RLR, RNR, RWR and RRR). MobileNet-based and VGG16-based autoencoders were applied on the Supervisely Person dataset. The results indicated that using rotation solely to augment data might not be a good for the segmentation task in this case.

Model	Baseline	RLR	TR	RCR	RNR	RWR	RRR
MobileNet	75.13±0.25	72.12±0.21	73.75±0.15	72.72±0.18	71.12±0.17	71.27±0.10	71.09±0.12
VGG16	75.42±0.16	72.56±0.19	73.16±0.22	72.66±0.24	71.31±0.08	71.22±0.14	71.06±0.15

**Table 7:** Segmentation accuracy comparison between different data augmentation techniques (i.e., TR, RCR, RLR, RNR, RWR and RRR). MobileNet-based and VGG16-based autoencoders were applied on the Nuclei images dataset. The results indicated that using rotation to augment data could enhance the segmentation performance in this case.

Model	Baseline	RLR	TR	RCR	RNR	RWR	RRR
MobileNet	94.25±0.05	97.6±0.08	95.37±0.11	94.50±0.28	95.28±0.19	95.24±0.14	95.43±0.13
VGG16	94.24±0.12	97.81±0.09	95.36±0.21	94.74±0.17	95.08±0.18	94.91±0.15	95.32±0.16

## Data Availability

The data presented in this study are available on request from the corresponding author.

## Abbreviations

The following abbreviations were used in this manuscript:

TR	Traditional rotation
RLR	Random local rotation
RCR	Random center rotation
